# Carpets vs. Health

**Published:** 1870-10

**Authors:** 


### CARPETS VS. HEALTH.

Years ago, in the days of our Grand Fathers and Grand Mothers—when the children in the family were numbered by tens and fifteens,—when more attention was paid to comfort and health than to show and dress,—when our Grand Mas did their own work and scrubbed their dining-room and kitchen floors with brush and sand,—when carpets were only used in the parlor,(if such a luxury was afforded) or in the "spare room," that was rarely occupied,—when the large fire place, with perfect ventilation, occupied one end of the living room;—in those good, old days, people lived healthfully, comfortably and sensibly, to a ripe old age, and rarely complained of disease. But now, in these days, when wives are oftener mistresses than mothers, and our girls are taught more how to dress and to make "a good match," than to make good, useful housekeepers and mothers, and when every room in the house is carpeted, so that we are compelled to breathe all manner of poisonous particles the moment you enter the door; it is quite evident that people are not as healthy, nor do they live as long, as in the days long ago.

There is no change in the manner of living from the days just mentioned, that has contributed more to the liability to disease, from indoor life, than the almost universal system of carpeting the floors. Not only are the floors of the parlor, sitting and bed rooms carpeted, but that of the dining room and quite frequently that of the kitchen, also. The habit is most pernicious to health. Apartments as constantly occupied as are the kitchen and dining room, always contain millions of particles of hair, cuticle, epithelium, ovules and fungi besides a large amount of other organic matter. These substances find a lodgment in our carpets and are set in motion by the skirts of our women and the romping of the children, until the air is literally alive with infection, which is taken into our nostrils and lungs to produce disease and shorten our lives. Just darken your dining-room some bright morning and admit a beam of sunlight through a crack or hole in the blind, stand at a right angle to the sun-beam, and observe the myriads of particles of dust flying through the light that before were unobserved —you will then probably not be surprised when we tell you that *such* air is unwholesome to breathe, that it contains infection and that disease will sooner or later result from its use.—These facts have been fully demonstrated by the use of the microscope in the hands of scientific men, amply competent to pronounce upon such subjects.

Let your kitchen and dining room floors remain bare. There are many ways of beautifying them without the use of carpets. A coat of paint is sufficient, or if something handsome is required, a floor laid in alternate strips of black-walnut and maple or oak is beautiful. Such floors can be scrubbed once or twice a week and mopped up regularly every morning, insuring to the family an absolutely clean room and pure air in which to enjoy their meals.

If such an arrangement could be extended to the sitting room it would be far more conducive to health, but in the kitchen and dining room it is absolutely necessary to good health and cleanliness. A good, hard-wood floor will cost no more than an ingrain carpet, will last ten times longer, is more cleanly, healthful and handsome, while a pine floor scrubbed with soap and sand until white, smooth and clean, is far handsomer than a dirty carpet and is within the reach of every family.

SEWAGE MANURING.—Experiments with sewage as manure have thus far proved entirely successful in England. At Barking, the fourth consecutive crop of wheat is now growing upon land treated in this way. The stalks are about five feet high, with ears of great length.

GENUINE PORT WINE.—Cider, 14 oz. Alcohol, 3 oz. Strong decoction of logwood, 4 oz. Alum, 40 grains. Cream of tartar, 20 grains.—White sugar, 1¼ oz. This, being a *native* wine, is largely patronized in America. By all means, make it yourself. It will be much cheaper than to buy it, and you will have the satisfaction of knowing that it is unadulterated.

